# Body Temperature Regulation in Hot Environments

**DOI:** 10.1371/journal.pone.0161481

**Published:** 2016-08-22

**Authors:** Jan-Åke Nilsson, Mary Ngozi Molokwu, Ola Olsson

**Affiliations:** 1 Department of Biology, Lund University, SE-223 62, Lund, Sweden; 2 Fauna & Flora International, 1000, Monrovia 10, Liberia; 3 A.P. Leventis Ornithological Research Institute, P.O. Box 13404, Jos, Nigeria; Università della Tuscia, ITALY

## Abstract

Organisms in hot environments will not be able to passively dissipate metabolically generated heat. Instead, they have to revert to evaporative cooling, a process that is energetically expensive and promotes excessive water loss. To alleviate these costs, birds in captivity let their body temperature increase, thereby entering a state of hyperthermia. Here we explore the use of hyperthermia in wild birds captured during the hot and dry season in central Nigeria. We found pronounced hyperthermia in several species with the highest body temperatures close to predicted lethal levels. Furthermore, birds let their body temperature increase in direct relation to ambient temperatures, increasing body temperature by 0.22°C for each degree of increased ambient temperature. Thus to offset the costs of thermoregulation in ambient temperatures above the upper critical temperature, birds are willing to let their body temperatures increase by up to 5°C above normal temperatures. This flexibility in body temperature may be an important mechanism for birds to adjust to predicted increasing ambient temperatures in the future.

## Introduction

Birds inhabit a wide range of thermal environments, posing problems to defend a constant core body temperature of about 41–42°C [1]. In hot environments the thermal gradient between body and environment may obstruct the transfer of excess heat formed during metabolism out of the body or even promote an influx of heat into the body at ambient temperatures above the thermoneutral zone [2]. To avoid overheating in such situations, birds actively dissipate heat from the body by increasing evaporative cooling, through panting and gular fluttering, although at least the former of these behaviours is connected to high metabolic costs [3]. Thus, food availability to cover increased energy expenditures is also important for birds in hot environments, however, water availability may be even more decisive for the potential to regulate body temperature as water is lost during evaporative cooling. Thus, for birds to avoid overheating at high ambient temperatures, water is lost through respiration and evaporation and this lost water needs to be replenished to keep the water balance. However, in arid regions and during the dry season in areas with dry-wet seasonality where water is in short supply, birds may allow body temperature to increase above normal, thus entering hyperthermia [4–6], reducing the total evaporative loss of water by up to 50% [4,7]. However, this body temperature flexibility to save water is size-dependent as its efficiency declines with body size, especially for long bouts of heat stress [4]. In large species (> 1 kg), the relation is even reversed with an increased water loss in hyperthermic birds [4]. Thus, large species are predicted to maintain normothermic body temperatures in hot environments to a much greater extent than small species [4,8].

As temperatures around the globe have increased substantially during the last decades [9] and are predicted to increase even more, the ability of organisms to deal with these high temperatures will be decisive for maintaining their distribution ranges [10]. This calls for behavioural and physiological flexibility such as adaptive hyperthermia [11,12] to reduce energy expenditures and conserve water [13].

Our knowledge about the use of hyperthermia to reduce energy costs and water loss in hot environments is, however confined to birds in laboratory settings [4] and data from wild birds are badly needed [2,12]. Here, we report on a study on core body temperatures in a seed-eating guild of small birds living in hot environments. We predict that as ambient temperature increases above the thermoneutral zone, birds become increasingly hyperthermic.

## Materials and Methods

The study was conducted at A. P. Leventis Ornithological Research Institute (APLORI), located in the Amurum Forest Reserve east of Jos, central Nigeria (09°53´N, 08°59´E). Located within the guinea savannah region, the area experiences a markedly seasonal climate with pronounced dry and wet seasons. The wet season starts around April or May and extends into early October while the dry season occurs from October to March [14]. The study was carried out between 9 and 14 of March 2009, i.e. during the hot and dry season with few remaining sources of water. The reserve consists of woodland savannah which includes grasslands with patches of shrubs and fringing forests along small streams that dry up during the dry season.

We operated 4–5 mist-nets, placed in the shade close to bushes or other presumed flyways, during two periods of the day; 06:45–11:00 and 15:00–18:00, respectively. When a bird got caught in a net, it was immediately extracted, placed in a cloth bag and had its body temperature measured within 5 min, with the majority of birds being measured within 2 min of capture. Body temperature was measured with a standard copper thermocouple (Ø 0.9 mm; ELFA AB, Järfälla, Sweden) connected to a Testo 925 digital thermometer (Testo AG, Lenzkirch, Germany). The digital thermometer was calibrated by Nordtec Instruments AB, Göteborg, Sweden (calibration certificate 128331 A) over a range of relevant temperatures (35–45°C). The thermocouple was inserted 12 mm into the cloaca and three readings were obtained within 10 s. Intra-sample repeatability was high (r = 0.99, P < 0.001; [15]) and the mean of the three readings were used in all analyses. The varying period of time that each individual spent in the cloth bag could potentially induce unwanted variation to the data-set. Birds may get stress-induced fever resulting in an increase in body temperature after handling or alternatively have time to dissipate some of their heat load resulting in a decrease in body temperature. Therefore, we looked for a possible trend in body temperature over time in the three body temperature readings taken for each individual. We found no significant trend (repeated measure ANOVA: F_1,68_ = 1.8; P = 0.18) in body temperature during this 10 s measuring period. In previous studies on handling stress in chickens (*Gallus domesticus*) and common eiders (*Somateria mollisima*) which have been suggested to induce fever, body temperature increased by 0.2–0.3°C and ca. 1°C, respectively, when measured 3 and 4 minutes after handling [16,17]. However, the induction of fever might be size dependent as a fever response to an infection is restricted to large birds, like the chicken, and not found in birds in the size class of those included in this study [18]. Furthermore, pied flycatchers (*Ficedula hypoleuca*), being in the same size class as the birds included in this study, had a very consistent body temperature as measured immediately after capture and again after 5–10 minutes in a cloth bag (repeatability: r = 0.85, P < 0.001) without showing any trend for increased or decreased temperature over this time period (paired-sample t-test: t = 1.23; df = 22; P = 0.23; A. Nord and J.-Å. Nilsson, unpublished). Therefore, as the stress-induced increase in body temperature at the most can only account for a small fraction of the temperature increase found in this study, we are confident that the variation in time between capture and measurement of individuals did not influence our conclusions.

After the body temperature measurement, we ringed the individual and measured mass (to the closest 0.1 g), tarsus length (to the closest 0.1 mm) and wing length (to the closest 0.5 mm). We measured ambient temperature with a small data logger (iButton DS1922, Maxim Integrated Products Inc., Sunnyvale, California) in the shade. The iButton logged the temperature every minute and the reading closest in time to the body temperature measurement were taken as the ambient temperature for that specific measurement.

As the advantage of hyperthermia may vary with body size [4,6], we restricted our sample of species to those with a mass below 20 g. In total, we captured 69 individuals of 13 species (Table 1). Six individuals were captured twice, although each individual was only used once in the analyses. In these cases the measurement at the highest ambient temperature were selected for the data-set.

**Table 1:** Species included in the analyses and their sample size and measured mass range. Nomenclature following Barrow and Demey [19].

Species	Sample size	Mass range (g)
Red-billed firefinch	23	7.4–10.5
*Lagonosticta senegala*		
Red-cheeked cordon-bleu	22	8.5–10.5
*Uraeginthus bengalus*		
Rock firefinch	7	9.8–12.0
*Lagonosticta sanguinodorsalis*		
Cinnamon-breasted rock bunting	4	12.8–15.7
*Emberiza tahapisi*		
Common whitethroat	3	14.1–16.1
*Sylvia communis*		
Lavender waxbill	2	9.7–10.3
*Estrilda caerulescens*		
Village indigobird	2	12.4–13.6
*Vidua chalybeata*		
Willow warbler	1	10.0
*Phylloscopus trochilus*		
Tawny-flanked prinia	1	8.5
*Prinia subflava*		
Rock-loving cisticola	1	13.6
*Cisticola aberrans*		
Yellow white-eye	1	9.4
*Zosterops senegalensis*		
Little weaver	1	15.4
*Ploceus luteolus*		
Bronze mannikin	1	9.7
*Spermestes cucullata*		

Data were analyzed using General Linear Models with body temperature as the dependent variable and tarsus length, wing length, mass, time of day and ambient temperature as independent variables. We also analysed the multi-species data-set using a mixed effects model with species as a random factor. However, as this model resulted in the same final model and had a marginally worse fit to the data (AIC: 212.6 vs 212.1), we only report the results from the General Linear Model. Full models were sequentially reduced by a backward stepwise elimination process, at each step removing the least significant variable, until only significant variables remained in the model. All statistical analyses were conducted in SAS Enterprise 4.3.

### Ethics statement

Permission to work in the field and approval of experimental procedures was granted by A. P. Leventis Ornithological Research Institute (APLORI), Jos, Nigeria. Nigeria has no formal ethical committees for animals in science but similar experimental procedures (with other species) for research in Sweden have been approved by the Malmö/Lund Ethical Committee (M 237–07). No endangered or protected species were involved in the study. No birds needed to be sacrificed in the study.

## Results

**Fig 1:**
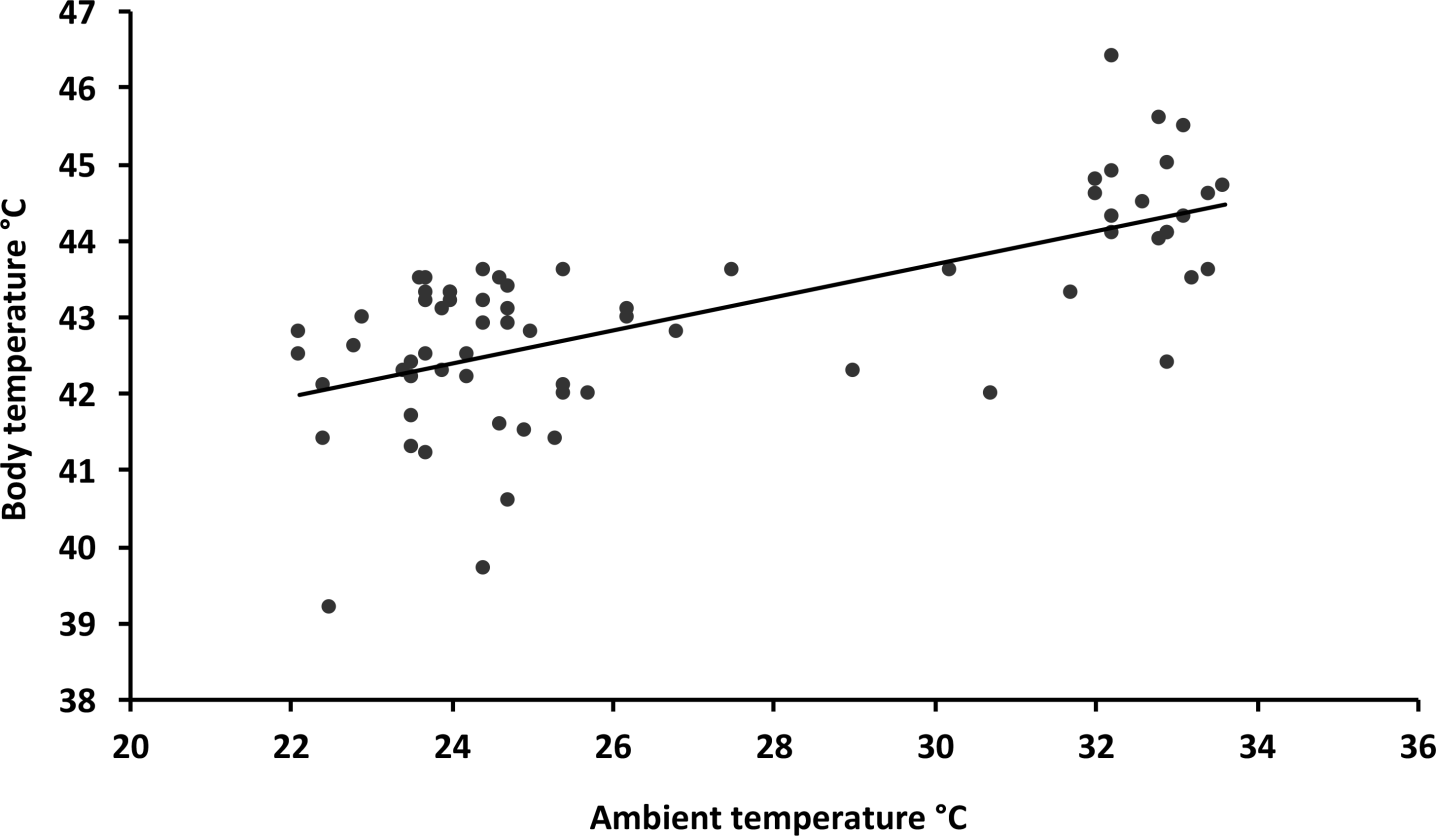
Relationship between ambient temperature in the shade and body temperature. Data from 69 individual birds from 13 different species. Equation of the line: Body temperature = 37.2 + 0.22(ambient temperature); R^2^ = 0.45.

**Fig 2:**
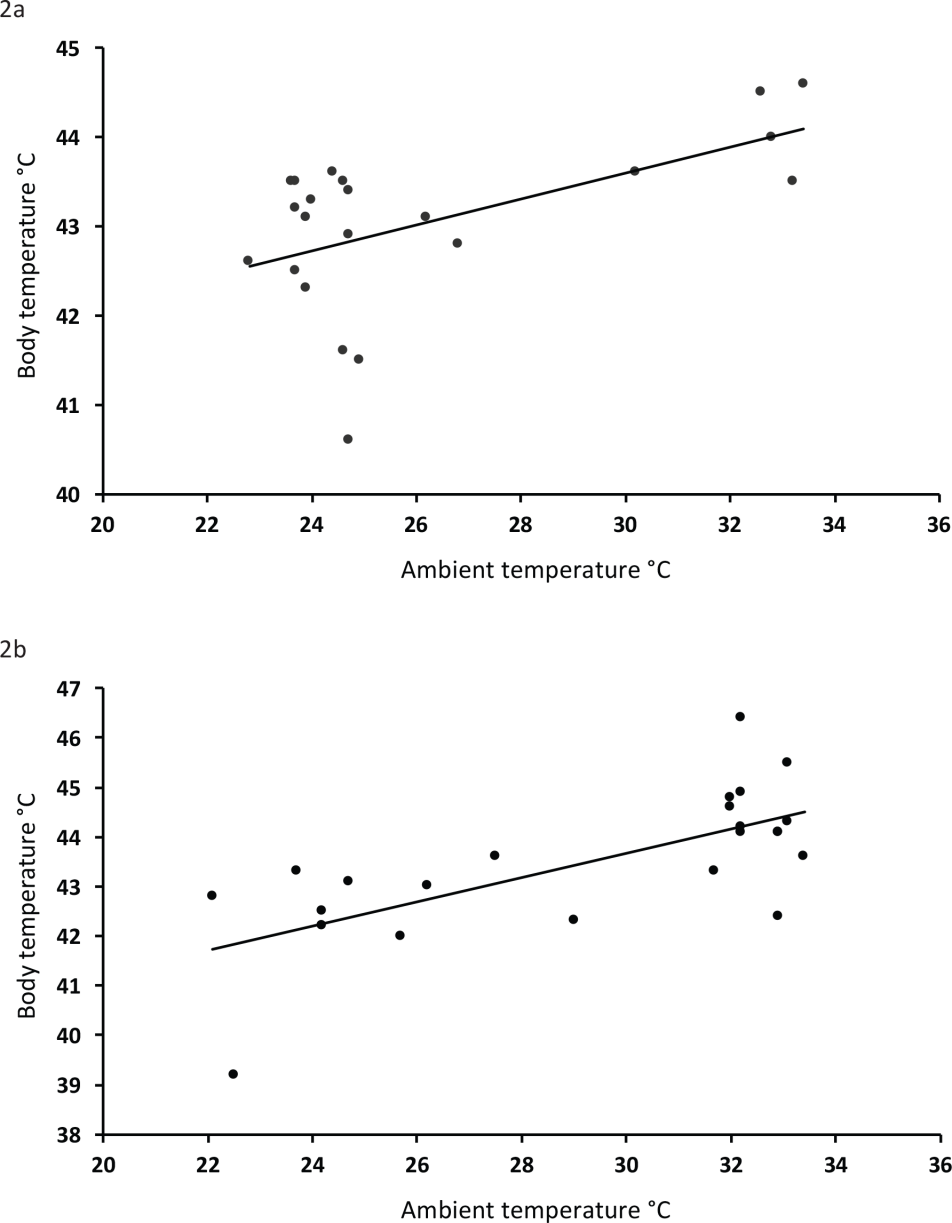
Relationship between ambient temperature in the shade and body temperature. Data from 22 individual red-cheeked cordon-bleus (A) and 23 individual red-billed firefinches (B). Equation of the line (A): Body temperature = 39.3 + 0.14(ambient temperature); R^2^ = 0.30, (B) Body temperature = 36.3 + 0.24(ambient temperature); R^2^ = 0.47.

Body mass varied between 7.4 and 16.1 g among individuals across all the species (Table 1), but none of the size/mass variables could explain any of the variation in body temperature in any of the analyses. Instead, body temperature was positively related to ambient temperature in the shade (F_1,67_ = 54.9; P < 0.001) with an increase in body temperature by 0.22°C for each degree increase in ambient temperature (Fig 1). To test for non-linear effects of ambient temperature, we also included the quadratic term of ambient temperature. Although also highly significant (P < 0.001), the fit of this model was worse than the linear model with only ambient temperature in explaining the variation (AIC: 206.9 vs 199.1). The highest body temperature measured during the study was 46.4°C in a red-billed firefinch (*Lagonosticta senegala*) and three other individuals (red-billed firefinch, village indigobird (*Vidua chalybeata*) and common whitethroat (*Sylvia communis*) had body temperatures exceeding 45°C. Sample size for two of the species, viz. red-cheeked cordon-bleu (*Uraeginthus bengalus*) and red-billed firefinch, was large enough to run the same analyses for these species separately. The results were the same as for the total data-set with a significant increase in body temperature with ambient temperature (red-cheeked cordon-bleu: F_1,20_ = 8.71; P = 0.008; red-billed firefinch: F_1,21_ = 18.8; P < 0.001; Fig 2).

## Discussion

We found that individuals from at least two wild bird species actually became hyperthermic in hot environments, presumably to reduce energy and water expenditures, and that the degree of hyperthermia is dependent on ambient temperature. We did, however, not find any relationship between body temperature and body size, stressing that the size span of our species is below the one where hyperthermia starts to have a negative impact on water retention ability [4,8]. The highest body temperatures recorded during the study was very close to predicted lethal levels of 46–47°C [20]. It should be noted though, that as we captured our birds in mist-nets, they had been flying immediately before body temperature measurements. Flying being a very energy demanding activity, thus generating much metabolic heat, may have resulted in a somewhat transient heat load. Flying pigeons (*Columba livia*) have been shown to increase their body temperature by 1–2°C above resting temperatures [21,22]. Although the resting body temperature is not known for the species included in this study, it is conceivable that part of the increased body temperature is related to flight. Birds living in hot environments usually have a somewhat higher upper critical temperature of the thermoneutral zone than species from cooler environments [23]. The average upper critical temperature of 28 passerine species living between latitudes 10°N and 10°S is 33.3°C (SD = 3.33°C; from supplementary material in [24]). Thus, resting in the shade in the ambient temperatures found in this study would in most cases allow for passive dissipation of such a transient heat load. However, irrespective of the reason for the increased body temperature in our study, it is evident that individuals have physiological pathways (e.g. in relation to protein stability and membrane fluidity) that can handle such high body temperatures even if it should be for only relatively short time periods.

Our results on wild birds comply with previous studies on birds in captivity. Reviewing these studies, Tieleman and Williams [4] found that at an ambient temperature of 45°C, all species became hyperthermic on average increasing their body temperature by 3.3°C. Furthermore, the rate of increase in body temperature with an increase in ambient temperature is comparable with studies in captivity [4,7], indicating the same mechanism for body temperature regulation in wild and captive birds. However, some of our wild birds have extremely high body temperatures not replicated in studies on captive birds. Thus, birds in the wild let their body temperatures rise higher than those in captivity which is not a surprise as several factors may be predicted to differ between wild and captive birds. Wild birds experience variation in ambient temperatures depending on if they reside in the shade or in direct sunlight. Captive birds are usually exposed to constant temperatures whereas wild, actively foraging birds have to move in and out of direct sunlight, considerably increasing their surface temperature above temperatures in the shade which are used as a reference in this study. Thus, free moving birds may accept a higher short-term heat load if this can later be passively dissipated due to a negative temperature gradient between the body and the environment by choosing sites in the shade for resting. Furthermore, as increasing body temperature in hot environments may be viewed as a trade-off between the use of hyperthermia and access to energy and water, the need for hyperthermia may be predicted to be less in captive birds with usually unrestricted access to food and water compared to wild birds. Previous studies of wild birds in the same study area has shown measurable costs of foraging exposed to the heat from direct sunlight, and the value of access to water [25]. It should, however, be noted that this study was conducted during the dry season and results would probably have been different if conducted during the wet season.

In endothermic animals, heat produced metabolically must be dissipated to avoid lethal body temperatures. Recently, it was suggested that the capacity to get rid of internally generated heat might constrain maximal energy expenditure, thereby constraining work rate and the set of possible life history strategies [26]. This constraint would be augmented in hot environments where experienced ambient temperatures may be similar or even higher than body temperatures restricting the possibilities for passive heat dissipation. This would be especially problematic for birds, as compared to mammals, as they have high metabolic rates and are to a greater extent diurnal, features making life in hot environments difficult. As a possible adaptation offsetting some of these problems, birds seem to be able to accept a wide range of body temperatures above normal temperatures (up to around 5°C; this study).

This flexibility in body temperatures may make birds well adapted to meet future global increases in ambient temperature [24,27]. However, environment-induced hyperthermia is probably connected to costs without being lethal. Increased production rates of reactive oxygen species (ROS) has been suggested as such a cost [11,28]. Along with the detrimental effects caused by ROS on essential molecules such as protein, lipids and DNA [29], hyperthermia has the potential to increase senescence and reduce life span. Furthermore, it has been shown that enzymes lose activity [30] and even that proteins degrade [31] in response to increased body temperatures. These costs may have to be traded-off to the benefits of work, which increase body temperature. In line with this, foraging efficiency was lower during hot days in an arid-zone bird species, resulting in failure to maintain body condition during such periods [32]. Obviously, the need for such trade-offs will potentially also have long-term consequences for survival.
